# High prevalence of colistin heteroresistance in specific species and lineages of *Enterobacter cloacae* complex derived from human clinical specimens

**DOI:** 10.1186/s12941-023-00610-1

**Published:** 2023-07-15

**Authors:** Shota Fukuzawa, Toyotaka Sato, Kotaro Aoki, Soh Yamamoto, Noriko Ogasawara, Chie Nakajima, Yasuhiko Suzuki, Motohiro Horiuchi, Satoshi Takahashi, Shin-ichi Yokota

**Affiliations:** 1 Department of Microbiology, Sapporo Medical University School of Medicine, Sapporo, Japan; 2 Clinical Laboratory, National Hospital Organization Hokkaido Cancer Center, Sapporo, Japan; 3 Laboratory of Veterinary Hygiene, Faculty of Veterinary Medicine, Hokkaido University, Sapporo, Japan; 4 Graduate School of Infectious Diseases, Hokkaido University, Sapporo, Japan; 5 One Health Research Center, Hokkaido University, Sapporo, Japan; 6 Department of Microbiology and Infectious Diseases, Toho University School of Medicine, Tokyo, Japan; 7 Division of Bioresources, Hokkaido University International Institute for Zoonosis Control, Sapporo, Japan; 8 International Collaboration Unit, Hokkaido University, International Institute for Zoonosis Control, Sapporo, Japan; 9 Institute for Vaccine Research and Development (HU-IVReD), Hokkaido University, Sapporo, Japan; 10 Department of Infection Control and Laboratory Medicine, Sapporo Medical University School of Medicine, Sapporo, Japan; 11 Division of Laboratory Medicine, Sapporo Medical University Hospital, Sapporo, Japan

**Keywords:** *Enterobacter cloacae* complex, Colistin, Heteroresistance, *arnT*, PhoPQ, Average nucleotide identity (ANI)

## Abstract

**Background:**

Colistin (CST) is a last-line drug for multidrug-resistant Gram-negative bacterial infections. CST-heteroresistant *Enterobacter cloacae* complex (ECC) has been isolated. However, integrated analysis of epidemiology and resistance mechanisms based on the complete ECC species identification has not been performed.

**Methods:**

Clinical isolates identified as “*E. cloacae* complex” by MALDI-TOF MS Biotyper Compass in a university hospital in Japan were analyzed. Minimum inhibitory concentrations of CST were determined by the broth microdilution method. The population analysis profiling (PAP) was performed for detecting the heteroresistant phenotype. The heat shock protein 60 (*hsp60*) cluster was determined from its partial nucleotide sequence. From the data of whole-genome sequencing, average nucleotide identity (ANI) for determining ECC species, multilocus sequence type, core genome single-nucleotide-polymorphism-based phylogenetic analysis were performed. *phoPQ-*, *eptA-,* and *arnT*-deleted mutants were established to evaluate the mechanism underlying colistin heteroresistance. The *arnT* mRNA expression levels were determined by reverse transcription quantitative PCR.

**Results:**

Thirty-eight CST-resistant isolates, all of which exhibited the heteroresistant phenotype by PAP, were found from 138 ECC clinical isolates (27.5%). The prevalence of CST-resistant isolates did not significantly differ among the origin of specimens (29.0%, 27.8%, and 20.2% for respiratory, urine, and blood specimens, respectively). *hsp60* clusters, core genome phylogeny, and ANI revealed that the CST-heteroresistant isolates were found in all or most of *Enterobacter roggenkampii* (*hsp60* cluster IV), *Enterobacter kobei* (cluster II), *Enterobacter chuandaensis* (clusters III and IX), and *Enterobacter cloacae* subspecies (clusters XI and XII)*.* No heteroresistant isolates were found in *Enterobacter hormaechei* subspecies (clusters VIII, VI, and III) and *Enterobacter ludwigii* (cluster V). CST-induced mRNA upregulation of *arnT*, which encodes 4-amino-4-deoxy-l-arabinose transferase, was observed in the CST-heteroresistant isolates, and it is mediated by *phoPQ* pathway. Isolates possessing *mcr-9* and *mcr-10* (3.6% and 5.6% of total ECC isolates, respectively) exhibited similar CST susceptibility and PAP compared with *mcr*-negative isolates.

**Conclusions:**

Significant prevalence (approximately 28%) of CST heteroresistance is observed in ECC clinical isolates, and they are accumulated in specific species and lineages. Heteroresistance is occurred by upregulation of *arnT* mRNA induced by CST. Acquisition of *mcr* genes contributes less to CST resistance in ECC.

**Supplementary Information:**

The online version contains supplementary material available at 10.1186/s12941-023-00610-1.

## Background

*Enterobacter cloacae* complex (ECC) and *Enterobacter aerogenes* (currently as *Klebsiella aerogenes*) represent the most frequently isolated *Enterobacter* spp. in respiratory, urinary tract, and bloodstream infections [1, 2]. According to recent literatures, following species, *Enterobacter asburiae*, *Enterobacter cancerogenus, Enterobacter cloacae*, *Enterobacter hormaechei*, *Enterobacter kobei*, *Enterobacter ludwigii*, *Enterobacter mori*, *Enterobacter nimipressuralis, Enterobacter roggenkampii,* and *Enterobacter soli* are assigned to ECC [3–5]. However, the classification has been expanding due to the application of whole-genome sequencing (WGS) to bacterial species identification. The clinical relevancy and antimicrobial resistance of individual ECC species are unclear because routine bacterial identification procedures, such as matrix-assisted laser desorption/ionization time-of-flight mass spectrometry (MALDI-TOF MS) and conventional biochemical testing with automated systems, used in clinical laboratories have not stay in step with classification of ECC into individual species [3]. Whereas heat shock protein 60 gene (*hsp60*) clustering have been used to classify ECC clinical isolates [6], DNA–DNA hybridization and average nucleotide identity (ANI) are the most reliable bacterial identifications at present [7].

Significant numbers of multidrug-resistant ECC have emerged [8, 9]. Thus, the choice of antimicrobials in the treatment of their infections appears to be becoming difficult. Colistin (CST) is a polycationic antimicrobial peptide that is effective and used as a last-line drug for multidrug-resistant Gram-negative bacteria infections, including ECC; however, CST-resistant ECC has emerged in clinical settings worldwide [6, 10, 11].

CST resistance is acquired by upregulation of the intrinsic lipid A-modifying enzyme gene, *eptA* (also known as *pmrC*) or *arnT* (present in the *arn* operon, *arnBCADTEF*), which encode the lipid A-modifying enzymes, phosphoethanolamine (pEtN) transferase and 4-amino-4-deoxy-l-arabinose (l-Ara4N) transferase, respectively [12]. Modification of lipid A decreases the negative charge of the outer membrane to repulse the positive charge of CST, leading to CST resistance [13]. Activation of two-component regulatory systems, PhoPQ and PmrAB, upregulates expression of the *eptA* and *arn* operon, and MgrB negatively regulates the two component system [12]. The mutations of *phoPQ, mgrB,* and/or *pmrAB* mediate CST resistance [14]. In addition, the worldwide spread of plasmid-mediated CST resistance genes, *mcr-1* to *mcr-10* has also been reported [15].

The CST resistance mechanism of ECC reported in the *E. cloacae* subsp. *cloacae* ATCC 13047 strain, which displays the CST-heteroresistant phenotype, involves the PhoPQ two-component system and *arn* operon [16]. CST heteroresistance refers to a bacterial clone containing both CST-susceptible and -resistant subpopulations, and causes therapeutic failure [17]. Thus, it necessitates assessment of CST-resistant subpopulations during clinical diagnosis of ECC infections.

In daily clinical laboratory investigations of antimicrobial susceptibility testing, the skip-well phenomenon with the broth microdilution method and the appearance of colonies in the inhibitory zone with the disc diffusion test and E-test are indications of the heteroresistant phenotype [11, 18]. Population analysis profiling (PAP) is the most reliable method to identify CST heteroresistance [19]. Unfortunately, identifications for ECC species and/or the CST heteroresistance in previous studies were inadequate [6, 10, 11, 16, 17].

This study aimed to elucidate the relationship between the genetic features and CST resistance mechanism by using the integrated analysis with molecular epidemiology [*hsp60* clustering, multilocus sequence typing (MLST), core genome analysis, and species identification by ANI] and the CST-heteroresistance [evaluations of skip-well phenomenon, PAP test, bacterial growth, and contributions of *phoPQ*, *mgrB*, *pmrAB*, *eptA*, and *arnT*] to comprehensively understand CST resistance in ECC clinical isolates.

## Methods

### Bacterial isolates

ECC isolates (n = 138) were derived from clinical specimens of different patients at Sapporo Medical University Hospital (Sapporo, Japan) from May 2017 to May 2018. These isolates were identified as “*E. cloacae* complex” by MALDI-TOF MS Biotyper Compass (software version 4.1.80.24), bacterial library version 8 (Bruker, Billerica, MA) in clinical laboratory testing. We also collected *K. aerogenes* (formerly, *E. aerogenes*) isolates (n = 30). These clinical specimens were respiratory (n = 78, 46.4%), urine (n = 22, 13.1%), blood (n = 12, 7.2%), and others (n = 56, 33.3%).

### Antimicrobial susceptibility testing, monitoring of bacterial growth, and detection of the skip-well phenomenon

CST, ampicillin, meropenem, gentamicin, and amikacin were purchased from FUJIFILM Wako Pure Chemical Corp. (Osaka, Japan). Cefotaxime and ciprofloxacin were purchased from Tokyo Chemical Industry (Tokyo, Japan). Minimum inhibitory concentration (MIC)s were determined by the broth microdilution method using cation-adjusted Mueller–Hinton II broth (MHII broth; Becton, Dickinson and Co., Franklin Lakes, NJ) according to the guidelines of the Clinical and Laboratory Standards Institute (CLSI) [20]. Isolates with a CST MIC of ≥ 4 mg/L were defined as CST-resistant according to CLSI M100-S32.

Bacterial growth was measured by assessing turbidity (OD_600_) after cultivation for 20 h under the same condition as used for the susceptibility test in the presence of CST (0–128 mg/L). Turbidity was measured using an Infinite M200 PRO instrument (Tecan, Kawasaki, Japan). The skip-well phenomenon was defined as no growth (OD_600_ < 0.1) at a certain CST concentration(s) and growth (OD_600_ > 0.5) at higher CST concentrations. Data are expressed as the means ± standard deviations from independent triplicate experiments.

### PAP

Heteroresistance was determined by PAP as previously described [19]. Briefly, ECC clinical isolates were inoculated into 10 mL of Trypticase Soy Broth (TSB) (Beckton, Dickinson and Co.) and cultured overnight at 37 ℃. The bacterial cell culture (approximately 1 × 10^9^ CFU/mL) was centrifuged and resuspended in 1 mL of sterile 0.85% NaCl. One hundred microliters of tenfold serial dilutions (from 1 × 10^10^ to 1 × 10^3^ CFU/mL) of the suspension were spread onto cation-adjusted Mueller–Hinton II agar (MHII agar; Becton, Dickinson and Co.) plates containing 0, 0.125, 1, or 8 mg/L CST and cultured for 20 h at 37 ℃. The occurrence frequencies of the CST-resistant subpopulations were calculated by dividing the numbers of CFU on CST-containing MHII agar plates by those on plain MHII agar plates. CST heteroresistance was determined using a previously published method based on the following criteria [19, 21]: (1) bacterial growth was confirmed in the presence of > 2 mg/L CST by the broth microdilution method, and (2) the CST concentration in the presence of which the CST-resistant subpopulations grew (within a frequency range from 1 × 10^–7^ to 1 × 10^–1^) were at least eightfold higher (≥ 1 mg/L CST) than the highest CST concentration that did not affect the growth of CST-susceptible subpopulations (0.125 mg/L CST). Data are expressed as the means ± standard deviations from three independent experiments.

### *hsp60* clustering

The 273 nucleotides of *hsp60* fragment was amplified by PCR with specific primers as described previously [22]. The nucleotide sequences of the *hsp60* fragment were aligned in MAFFT [23] (http://www.ebi.ac.uk/Tools/msa/mafft/). The phylogenetic tree was generated by MEGA X [24].

### WGS

To determine the draft whole-genome sequences of 95 ECC clinical isolates (Table 1), genomic DNA was extracted from bacteria using DNeasy Blood & Tissue Kit (Qiagen, Hulsterweg, The Netherlands), and DNA library for WGS was prepared using Nextera XT DNA Library Preparation Kit (Illumina, San Diego, CA) according to the manufacturer’s instructions. The libraries were sequenced on MiSeq Sequencing System (Illumina) with 300 bp paired-end reads according to the manufacturer’s protocol.

**Table 1:** Species determined by ANI, *hsp60* cluster classification, and CST susceptibility of ECC clinical isolates

*hsp60* cluster	ECC species (No. of isolates)	
	CST heteroresistant (38)	CST susceptible (57)
I	*E. asburiae* (7), *E. cancerogenus* (1)	*E. asburiae* (16), *E. soli* (1)
II	*E. kobei* (14)	*E. kobei* (1)
III	*E. chuandaensis*^a^ (1)	*E. hormaechei* subsp. *hoffmannii* (2)
IV	*E. roggenkampii* (9)	–
V	–	*E. ludwigii* (15)
VI	–	*E. hormaechei* subsp. *xiangfangensis* (6), *E. hormaechei* subsp. *oharae* (1)
VIII	–	*E. hormaechei* subsp. *steigerwaltii* (13)
IX	*E. chuandaensis*^a^ (3)	*E. chuandaensis*^a^ (1)
XI	*E. cloacae* subsp. *cloacae* (1)	–
XII	*E. cloacae* subsp. *dissolvens* (2)	–
XIII	–	*E. mori* (1)

^a^ANI value 94.1%

### MLST

MLST was performed using ECC MLST databases in PubMLST.org (https://pubmlst.org/organisms/enterobacter-cloacae/) and the Center for Genomic Epidemiology MLST 2.0 Web tool (https://cge.cbs.dtu.dk/services/MLST/).

### ECC species identification

ECC species were identified by ANI analysis using fastANI of the draft WGS data compared with the type strain genomes obtained from GenBank [25]. A cutoff of 98% of the ANI value compared with the type strains was used for species and subspecies identification [7, 26].

### Phylogenetic analysis of core genome single nucleotide polymorphisms (SNPs), annotation, and *mcr* gene identification

Core genome SNPs-based phylogenetic analysis was performed with WGS data. MiSeq read data were mapped to the reference genome, *E. cloacae* ATCC 13047 (accession number: NC_014121.1), using bwa version 0.7.17. The core genome of the applied strain was extracted using mpileup of SAMtools version 1.9 and the “cns” option of VarScan version 2.3.9 [27, 28]. A phylogenetic tree based on SNPs in the core genome was generated using the default parameter of FastTree version 2.1.10 [29]. Genome annotation was performed with DFAST v1.1.0 and standard settings [30] (https://doi.org/10.1093/bioinformatics/btx713). DNA sequences were compared using Clustal W [31] (https://www.genome.jp/tools-bin/clustalw). *mcr* genes were identified using assembled genome data by Resfinder (https://www.genomicepidemiology.org).

### Reverse transcription quantitative PCR (RT-qPCR)

Overnight cultures with TSB were diluted 1:100 in MHII broth, and cultivated at 37 ℃ for 0, 5, 15, 30, 60, and 120 min in the presence of CST (0, 0.25, 1, 4, 16, and 64 mg/L). Total RNA was extracted using a RNeasy Plus Mini Kit (Qiagen). cDNA was synthesized from total RNA (100 ng) using ReverTra Ace RT-qPCR Master Mix with Genomic DNA (gDNA) Remover (Toyobo). RT-qPCR was performed on LightCycler 480 II system (Roche, Mannheim, Germany) using KOD SYBR qPCR Mix (Toyobo) and primer pairs for *arnT*, which are shown in Additional file 1: Table S1. Gene expression levels relative to the housekeeping gene *gyrB* were determined using the ΔΔCt method. Data are expressed as the means ± standard deviations from three independent experiments.

### Construction of *phoPQ*-, *eptA*-, and *arnT*-deleted mutants

Deletion mutants of *phoPQ, eptA*, and *arnT* were generated by the λ red recombinase method using pKD46-hyg [32, 33]. Mini-cassette genes containing 50 bases corresponding to the upstream and downstream regions of the target genes and kanamycin resistance cassettes (Gene Bridges, Heidelberg, Germany) were amplified by PCR and the gene deletion was confirmed by PCR using specific primers (Additional file 1: Table S1).

### Statistical analysis

Mann–Whitney and a one-way ANOVA with the Kruskal–Wallis tests were used to examine the significance of differences between two and multiple groups, respectively. Occurrence rates of phenotypes among groups were examined by χ^2^-test. *p* < 0.05 was considered significant. Pearson’s correlation coefficients were also calculated.

## Results

### Classification of ECC and *K. aerogenes* clinical isolates into *hsp60* clusters

**Fig. 1:**
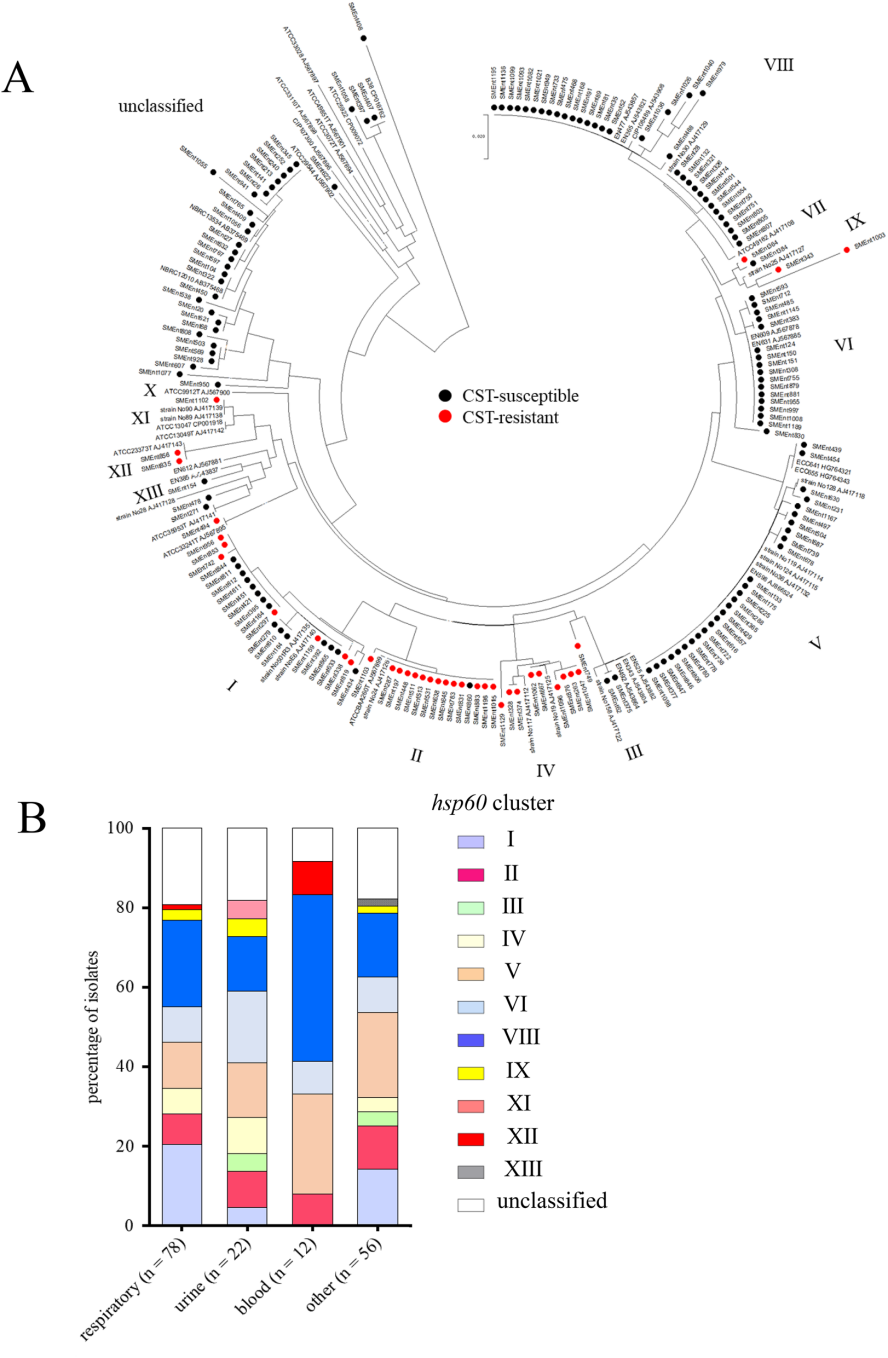
Classification of ECC and *K. aerogenes* into *hsp60* clusters. **A** Neighbor-joining unrooted tree based on *hsp60* clustering of ECC and *K. aerogenes* clinical isolates. CST-resistant and -susceptible isolates are indicated by red and black circles, respectively. The GenBank accession numbers of the reference genes are AJ417128 (I), AJ417141 (I), AJ567895 (I), AJ417135 (I), AJ417140 (I), AJ567899 (II), AJ417126 (II), AJ543882 (III), AJ543804 (III), AJ543864 (III), AJ417112 (IV), AJ417125 (IV), AJ866524 (V), AJ417132 (V), AJ417115 (V), AJ417114 (V), AJ417118 (V), HG764321 (V), HG764343 (V), AJ567878 (VI), AJ567885 (VI), AJ417108 (VII), AJ543857 (VIII), AJ543908 (VIII), AJ543821 (VIII), AJ417127 (IX), AJ567900 (X), AJ417138 (XI), AJ417139 (XI), CP001918 (XI), AJ417142 (XI), AJ417143 (XII), AJ567881 (XIII), AJ543837 (XIII), CP016762 (unclassified), CP009072 (unclassified), AJ567897 (unclassified), AJ567901 (unclassified), AJ567902 (unclassified), AJ567894 (unclassified), AJ567898 (unclassified), AJ567896 (unclassified), AB375469 (unclassified), and AB375468 (unclassified). **B** Comparison of *hsp60* cluster types of ECC and *K. aerogenes* clinical isolates according to the origin of clinical specimens. Significant differences of the occurrence rates of *hsp60* clusters did not observe among the origins of specimens

We analyzed ECC isolates and *K*. *aerogenes* isolates. Because *K*. *aerogenes* was formerly classified in genus *Enterobacter*, and CST-resistant *K*. *aerogenes* was reported [3, 11]. The 138 ECC isolates were classified into 11 *hsp60* clusters, and 30 *K. aerogenes* isolates were defined as unclassified (Fig. 1A, Additional file 1: Table S2). The most abundant cluster was VIII, followed by V, I, and VI (Table 2). There was no significant bias in the distribution of *hsp60* cluster types according to the origin of the clinical specimens (Fig. 1B).

**Table 2:** *hsp60* cluster classification and prevalence of CST-resistant ECC clinical isolates

*hsp60* cluster	No. of ECC isolates (percentage among ECC isolates)	No. of CST-resistant isolates (percentage among each cluster)
I	25 (18.1%)	8 (32.0%)
II	15 (10.9%)	14 (93.3%)
III	3 (2.2%)	1 (33.3%)
IV	9 (6.5%)	9 (100%)
V	27 (19.6%)	0 (0%)
VI	17 (12.3%)	0 (0%)
VIII	34 (24.6%)	0 (0%)
IX	4 (2.9%)	3 (75.0%)
XI	1 (0.7%)	1 (100%)
XII	2 (1.4%)	2 (100%)
XIII	1 (0.7%)	0 (0%)

### CST susceptibility of ECC and *K. aerogenes*, and its relationships with the origin of the clinical specimens and *hsp60* clustering

The MIC of CST was determined for the 138 ECC and 30 *K. aerogenes* clinical isolates (Table 3). It ranged from 0.5 to > 128 mg/L, and MIC_50_ and MIC_90_ were 1 and > 128 mg/L, respectively. Thirty-eight (27.5%) of ECC isolates were CST-resistant (MIC ≥ 8 mg/L), whereas none of CST-resistant isolates were observed in *K. aerogenes*.

**Table 3:** CST susceptibility testing of 138 ECC and 30 *K*. *aerogenes* clinical isolates

Bacterial species	CST MIC (mg/ L)												
	0	0.125	0.25	0.5	1	2	4	8	16	32	64	128	> 128
No. of ECC isolates	–	–	–	44	56	–	–	1	2 (2)	2 (1)	–	5	28 (7)
No. of *K. aerogenes* isolates	–	–	–	18	12	–	–	–	–	–	–	–	–

Breakpoint, 2 mg/L of CST. Parentheses indicates No. of isolates displaying the skip-well phenomenon

**Fig. 2:**
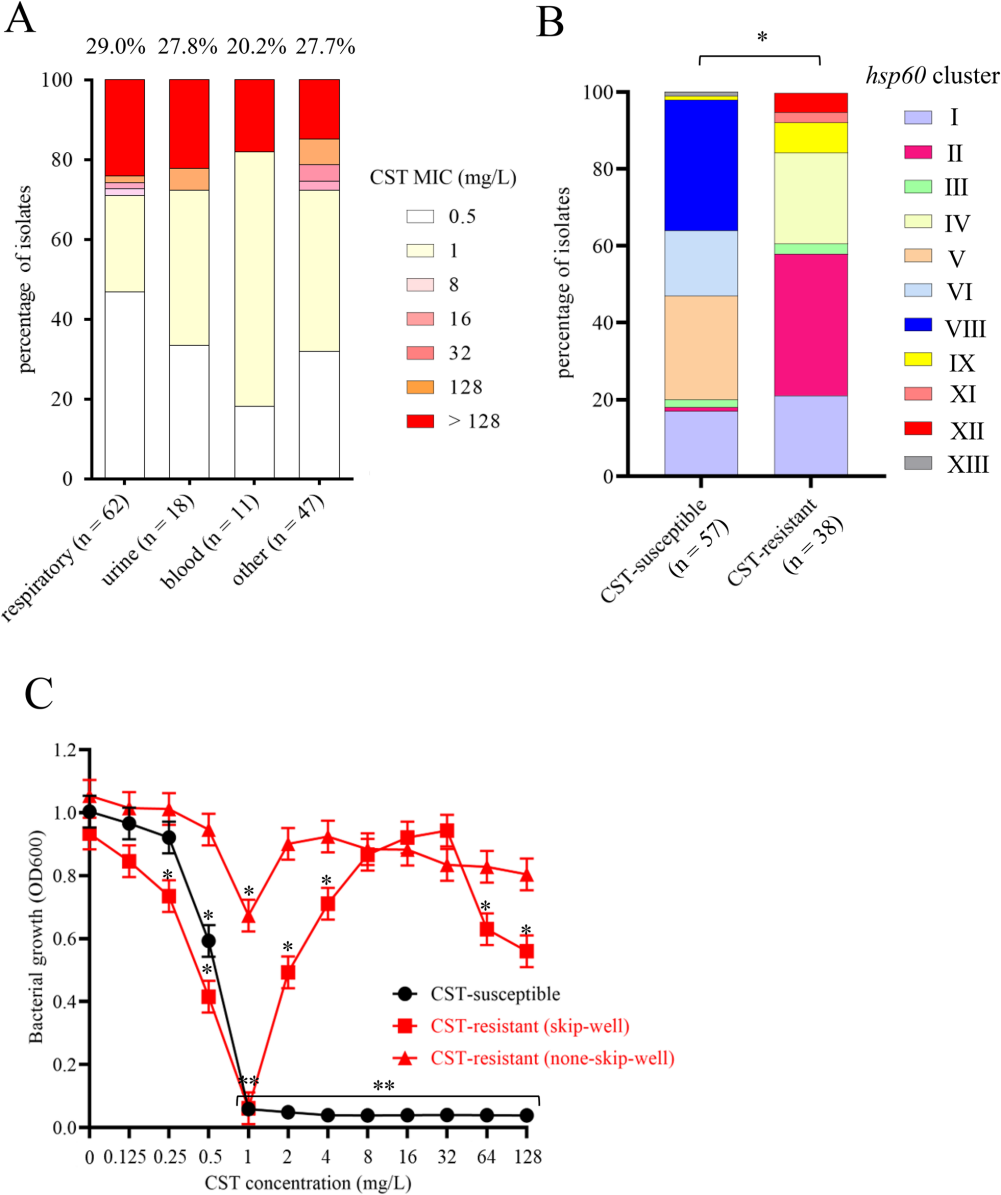
Comparison of CST-resistant and -susceptible ECC. **A** Comparison of CST MICs of ECC clinical isolates according to the origin of clinical specimens. Percentages indicate the prevalence of CST-resistant isolates. Numbers in parentheses indicate the number of ECC isolates. CST susceptibility did not significantly differ according to the origin of clinical isolates. **B** *hsp60* cluster classification of CST-susceptible and -resistant ECC clinical isolates. Significant difference was observed between the groups (*p < 0.05). **C** Growth of CST-susceptible and -resistant (with and without the skip-well phenomenon) ECC clinical isolates in the presence of various CST concentrations. Bacterial growth was measured by assessing turbidity (OD_600_) after cultivation for 20 h based on the broth microdilution method in 96-well plates. The skip-well phenomenon was defined as no growth (OD_600_ < 0.1) at a certain CST concentration(s) and growth (OD_600_ > 0.1) at higher CST concentrations. Representative ECC clinical isolate data are shown: CST-susceptible isolate (SMEnt52 strain; *E. hormaechei* subsp. *steigerwaltii*, *hsp60* cluster VIII), CST-resistant isolate displaying the skip-well phenomenon (SMEnt513 strain; *E. kobei*, *hsp60* cluster II), and CST-resistant isolate not displaying the skip-well phenomenon (none-skip-well) (SMEnt1102 strain; *E. cloacae* subsp. *cloacae*, *hsp60* cluster XI). Bacterial growths (OD_600_ values) in the presence of CST were compared with the bacterial growth in the absence of CST of the respective strains (*p < 0.05 and **p < 0.001)

The prevalence of CST-resistant ECC isolates did not significantly differ according to the origin of the clinical specimens (Fig. 2A). The distribution of *hsp60* cluster types significantly differed between CST-susceptible and -resistant isolates (*p* < 0.01) (Fig. 2B). CST-resistant isolates comprised seven clusters, and clusters II, IV, and I were dominant (Table 2). All isolates in clusters IV, XI, and XII, almost all isolates in clusters II and IX, and approximately one-third of isolates in clusters I and III were CST-resistant.

### Skip-well phenomenon and PAP

Ten CST-resistant isolates exhibited the skip-well phenomenon (26.3% of CST-resistant isolates and 7.2% of total ECC isolates) (Table 3, Additional file 1: Table S2). Their growth was almost completely inhibited in wells containing 0.5 or 1 mg/L CST, but cells grew in wells containing higher CST concentrations (higher than 2 mg/L) (Fig. 2C). Although the remaining 28 CST-resistant isolates did not display the skip-well phenomenon, they exhibited weak growth (OD_600_ of 0.2–0.6) in wells containing 0.5 or 1 mg/L CST and strong growth (OD_600_ > 0.8) in wells containing higher CST concentrations (≥ 2 mg/L) (Fig. 2C).

**Fig. 3:**
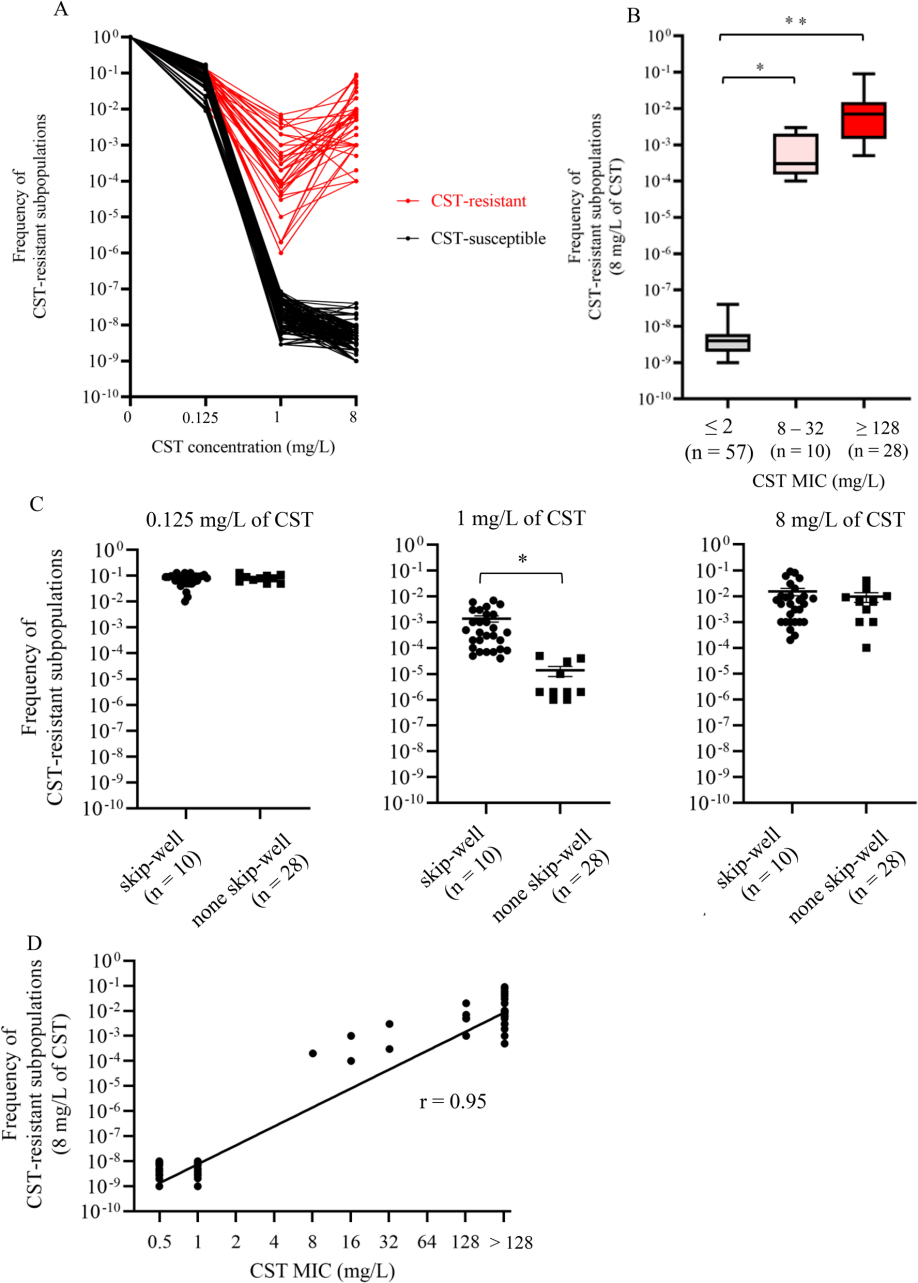
PAP of ECC. **A** Occurrence frequencies of CST-resistant subpopulations in the presence of various CST concentrations (0, 0.125, 1, and 8 mg/L). **B** Occurrence frequencies of CST-resistant subpopulations in isolates with low, moderate, and high CST MICs. Number in parentheses indicates the number of ECC isolates. *p < 0.05 and **p < 0.001. **C** Comparison of the occurrence frequencies of CST-resistant subpopulations between CST-resistant isolates displaying and not displaying the skip-well phenomenon in the presence of 0.125, 1, and 8 mg/L CST. Number in parentheses indicates the number of ECC isolates. *p < 0.05. **D** Correlation between CST MICs and occurrence frequencies of CST-resistant subpopulations

To analyze the CST-heteroresistant phenotype by PAP, we measured the occurrence frequencies of CST-resistant subpopulations in the presence of various CST concentrations (Fig. 3A). Growth of all isolates was unaffected in the presence of 0.125 mg/L CST. For all CST-susceptible isolates, the occurrence frequency of CST-resistant subpopulations was lower than 1 × 10^–7^ (ranged from 1.6 × 10^–9^ to 8.5 × 10^–8^) in the presence of 1 and 8 mg/L CST. For all CST-resistant isolates, the occurrence frequency of CST-resistant subpopulations in the presence of 1 and 8 mg/L CST (more than eightfold higher than 0.125 mg/L) exceeded 1 × 10^–7^ (ranged from 2.2 × 10^–6^ to 7.1 × 10^–3^ at 1 mg/L and from 1.3 × 10^–4^ to 6.4 × 10^–2^ at 8 mg/L). These results indicate that all CST-resistant ECC clinical isolates were CST heteroresistant.

The occurrence frequencies of CST-resistant subpopulations in CST-resistant isolates with high (≥ 128 mg/L) and moderate (8–32 mg/L) MICs were significantly higher than those in CST-susceptible isolates (Fig. 3B). They also tended to be higher in CST-resistant isolates with high MICs than in those with moderate MICs, but this difference was not significant. The occurrence frequencies of CST-resistant subpopulations in the presence of 1 mg/L CST were significantly higher in CST-resistant isolates that did not exhibit the skip-well phenomenon than in those that did (*p* < 0.05) (Fig. 3C), whereas there is no significant differences in 0.125 and 8 mg/L of CST. The occurrence frequencies of CST-resistant subpopulations correlated with the CST MICs (r = 0.95) (Fig. 3D).

**Fig. 4:**
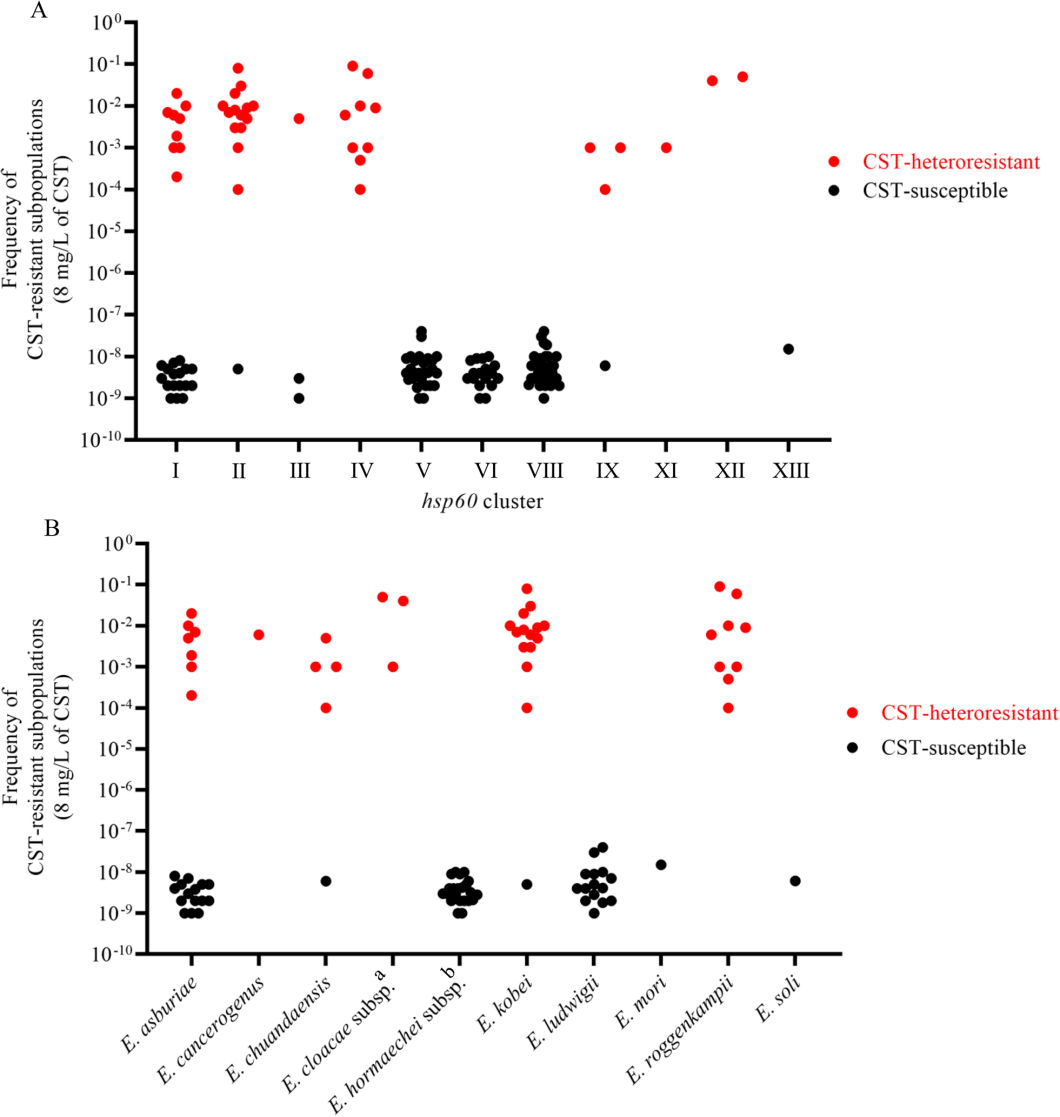
ECC species and occurrence frequencies of CST-resistant subpopulations. **A** Occurrence frequencies of CST-resistant subpopulations according to *hsp60* cluster classification. **B** Occurrence frequencies of CST-resistant subpopulations according to ECC species determined by ANI. CST-heteroresistant and -susceptible isolates are indicated by red and black circles, respectively. ^a^*E. cloacae* subsp. including *E. cloacae* subsp. *cloacae* (n = 1) and *E. cloacae* subsp. *dissolvens* (n = 2). ^b^*E. hormaechei* subsp. including *E. hormaechei* subsp. *hoffmannii* (n = 2), *E. hormaechei* subsp. *oharae* (n = 1), *E. hormaechei* subsp. *steigerwaltii* (n = 13), and *E. hormaechei* subsp. *xiangfangensis* (n = 6)

We evaluated the relationship between the occurrence frequencies of CST-resistant subpopulations and *hsp60* cluster types. In agreement with the results of CST susceptibility testing (Table 3), CST-heteroresistant isolates with higher occurrence frequencies of CST-resistant subpopulations (> 1 × 10^–5^) were found in certain *hsp60* clusters (I, II, III, IV, IX, XI, and XII) but not in other *hsp60* clusters (*p* < 0.001) (Fig. 4A), and in certain species, such as *E. kobei*, *E. roggenkampii*, and *E. cloacae* subsp. (*p* < 0.001) (Fig. 4B).

### MLST analysis and antimicrobial susceptibilities of CST-heteroresistant ECC clinical isolates

MLST analysis of the CST-heteroresistant isolates was performed. Sequence type (ST)s, ST1939, ST125, ST997, and ST1953 were the dominant in *hsp60* clusters I, II, IV, and XII, respectively. In antimicrobial susceptibility testing, 12 (31.6%) CST-heteroresistant isolates were resistant to cefotaxime and one was resistant to meropenem (Additional file 1: Table S3). No CST-heteroresistant isolate was resistant to aminoglycosides and ciprofloxacin.

### ECC species identification by ANI and generation of a phylogenetic tree based on core genome SNPs

**Fig. 5:**
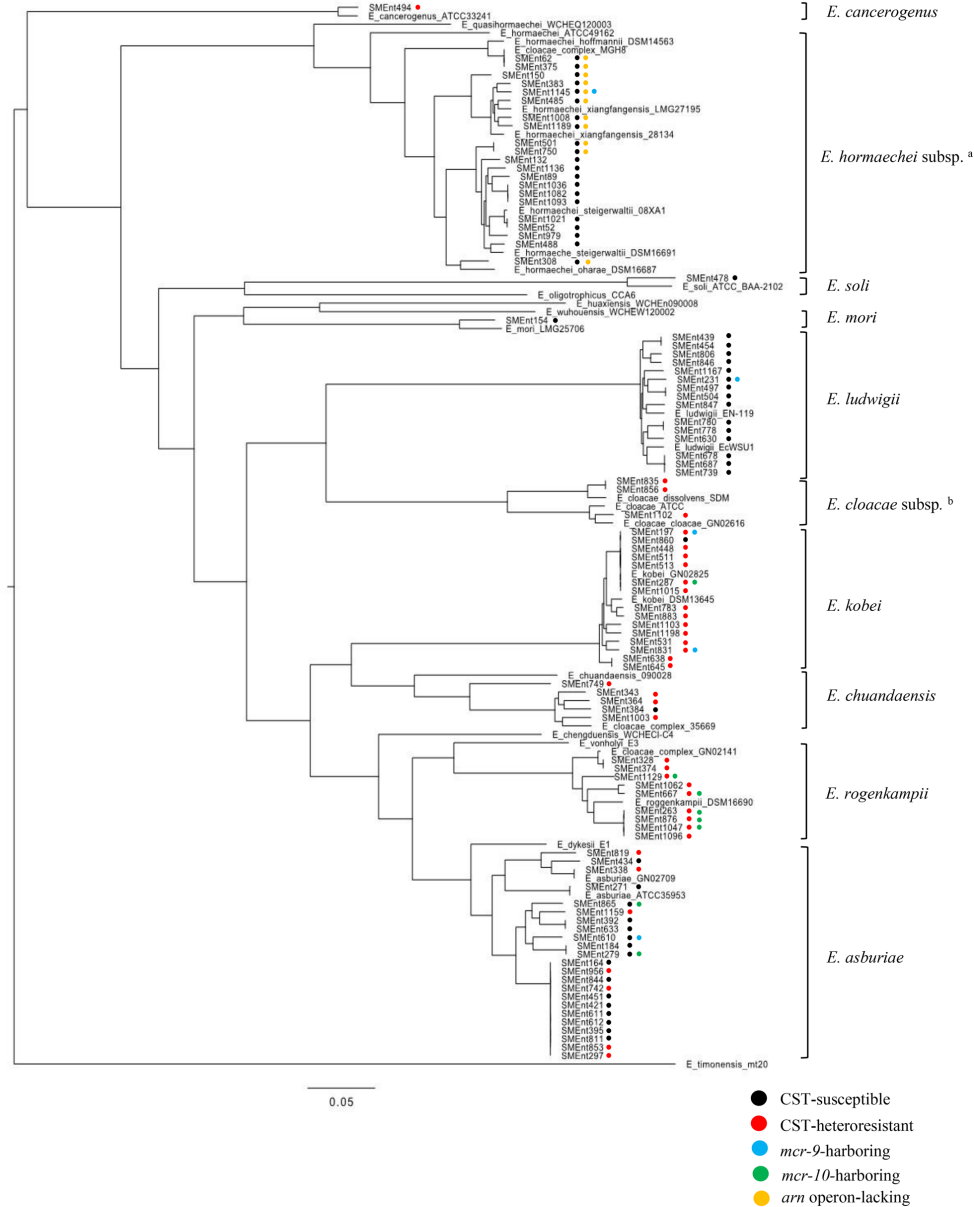
Phylogenetic tree based on core genome SNPs of ECC. Core genome SNP-based phylogenetic analysis was performed with WGS data of 95 ECC clinical isolates. CST-susceptible strains, CST-heteroresistant strains, strains harboring *mcr-9*, strains harboring *mcr-10*, and strains lacking the *arn* operon are indicated by black, red, blue, green, and yellow circles to the right of their names. ^a^*E. hormaechei* (subsp. *hoffmannii,* subsp. *xiangfangensis,* subsp. *steigerwaltii*, and subsp. *oharae*). ^b^*E. cloacae* (subsp. *cloacae* and subsp. *dissolvens*). The GenBank accession numbers of the reference genes are NZ_CP011863 (*E. asburiae*), NZ_LEDH00000000 (*E. asburiae*), NZ_FYBA00000000 (*E. cancerogenus*), NZ_CP043318 (*E. chengduensis*), NZ_QZCS00000000 (*E. chuandaensis*), NC_014121 (*E. cloacae* subsp. *cloacae*), NZ_LEDL00000000 (*E. cloacae* subsp. *cloacae*), NC_018079 (*E. cloacae* subsp. *dissolvens*), NZ_VTTY00000000 (*E. dykesii*), NZ_CP017186 (*E. hormaechei* subsp. *hoffmannii*), NZ_AFHR01000000 (*E. hormaechei* subsp. *hormaechei*), NZ_CP017180 (*E. hormaechei* subsp. *oharae*), NZ_CP017179 (*E. hormaechei* subsp. *steigerwaltii*), NZ_AMGJ01000000 (*E. hormaechei* subsp. *steigerwaltii*), NZ_CP017183 (*E. hormaechei* subsp. *xiangfangensis*), NZ_LABA01000000 (*E. hormaechei* subsp. *xiangfangensis*), NZ_CP043342 (*E. huaxiensis*), NZ_CP01718 (*E. kobei*), *NZ_LEDC01000000* (*E. kobei*), NZ_JTLO00000000 (*E. ludwigii*), NC_016514 (*E. ludwigii*), NZ_GL890773 (*E. mori*), NZ_AP019007 (*E. oligotrophicus*), NZ_SJON00000000 (*E. quasihormaechei*), NZ_CP017184 (*E. roggenkampii*), NZ_LXES00000000 (*E. soli*), NZ_FCOP00000000 (*E. timonensis*), NZ_VTUC00000000 (*E. vonholyi*), NZ_SJOO00000000 (*E. wuhouensis*), NZ_JZZB01000000 (ECC), NZ_LEEP01000000 (ECC), and NZ_AXLJ01000000 (ECC)

Ninety-five clinical isolates identified as ECC by the MALDI Biotyper were analyzed by ANI, and a phylogenetic tree was generated using core genome SNPs (Fig. 5, Additional file 1: Table S2). All 38 CST-heteroresistant isolates and 57 CST-susceptible isolates randomly selected to include half the isolates in each *hsp60* cluster were examined. Ten species were identified by ANI (Additional file 1: Table S2). The CST-heteroresistant isolates were identified as one of six species: *E. asburiae*, *E. cancerogenus, E. chuandaensis, E. cloacae* (including subsp. *cloacae* and subsp. *dissolvens*), *E. kobei*, and *E. roggenkampii* (Table 1). The 57 CST-susceptible isolates were identified as one of seven species: *E. asburiae, E. chuandaensis, E. hormaechei* (including subsp. *hoffmannii*, subsp. *oharae,* subsp. *steigerwaltii*, and subsp. *xiangfangensis*), *E. kobei*, *E. ludwigii, E. mori*, and *E. soli* (Table 1). The occurrence frequencies of CST-resistant subpopulations were closely related with the results of species identification, phylogeny, and *hsp60* clustering (Fig. 4A, B).

In CST-susceptible ECC, 10 isolates of *E. hormaechei* subsp. (including subsp. *hoffmannii*, subsp. *oharae*, subsp. *steigerwaltii*, and subsp. *xiangfangensis*) lacked the *arn* operon in their genomes according to annotation analysis, whereas other chromosomal CST resistance determinants (*phoPQ, mgrB, pmrAB,* and *eptA*) were present (Fig. 5). *mcr-9* and *mcr-10* were detected in five isolates (two *E. kobei* and one each of *E. asburiae*, *E. hormaechei* subsp. *xiangfangensis*, and *E. ludwigii*) and eight isolates (five *E. roggenkampii*, two *E. asburiae*, and one *E. kobei*), respectively (Fig. 5). The CST MIC and occurrence frequency of CST-resistant subpopulations analyzed by PAP did not significantly differ among the none-*mcr*, *mcr-9*-positive, and *mcr-10*-positive groups (Additional file 1: Table S4, Additional file 2: Fig. S1).

### *arnT* mRNA expression levels in CST-heteroresistant and -susceptible ECC clinical isolates

**Fig. 6:**
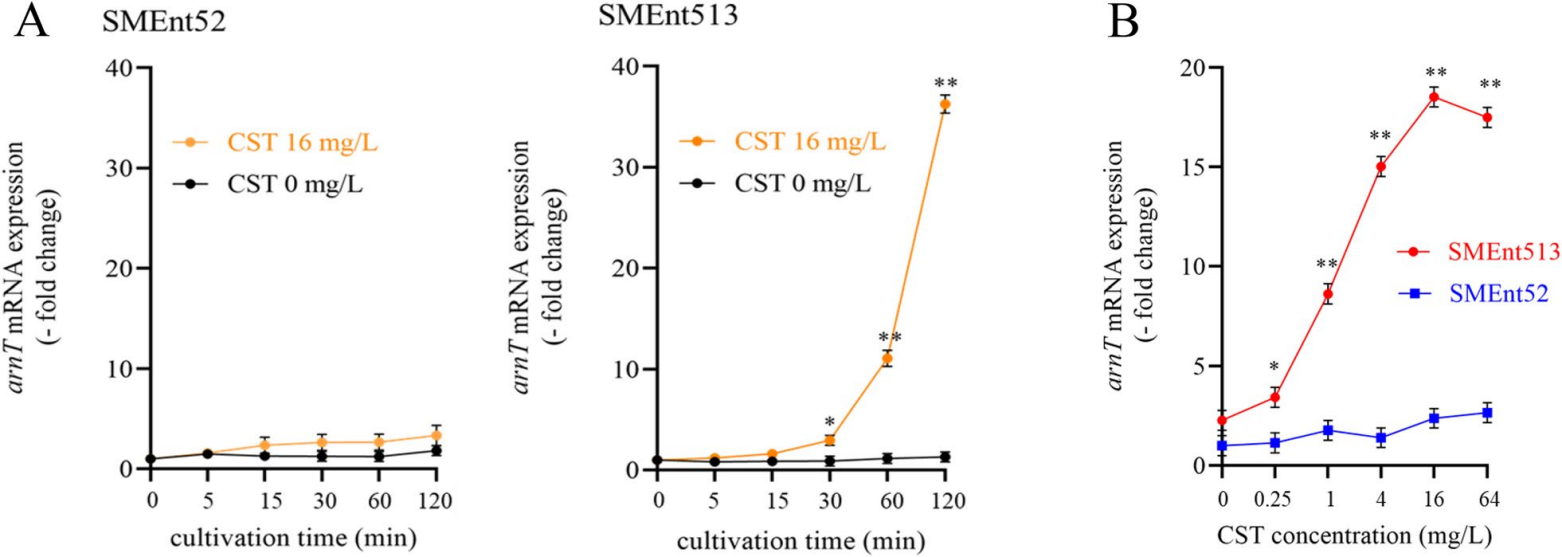
*arnT* mRNA expression levels in CST-susceptible and -heteroresistant ECC. SMEnt52 (*E. hormaechei* subsp. *steigerwaltii*, *hsp60* cluster VIII) and SMEnt513 (*E. kobei*, *hsp60* cluster II) were used as CST-susceptible and -resistant isolates, respectively. **A** *arnT* mRNA expression levels during cultivation of SMEnt52 and SMEnt513 in the presence (16 mg/L) and absence of CST. The y axis shows relative expression levels normalized by that at 0 min (set to 1.0). *p < 0.05 and **p < 0.001. **B** *arnT* mRNA expression levels in SMEnt52 and SMEnt513 cultivated in the presence of various CST concentrations. Total RNA was isolated after cultivation for 45 min at 37 ℃. The y axis shows relative mRNA expression levels normalized by that in SMEnt52 in the absence of CST (set to 1.0). *p < 0.05 and **p < 0.001

We measured the *arnT* mRNA expression levels in the CST-heteroresistant strain SMEnt513 (*E. kobei*, *hsp60* cluster II which mostly includes CST-heteroresistant isolates) and the CST-susceptible strain SMEnt52 (*E. hormaechei* subsp. *steigerwaltii*, *hsp60* cluster VIII which mostly includes CST susceptible isolates) in the presence and absence of CST. CST induced *arnT* mRNA expression in SMEnt513, but not in SMEnt52 (Fig. 6A). *arnT* mRNA expression in SMEnt513 was significantly increased by treatment with 0.25 mg/L CST and peaked (approximately 18-fold) upon treatment with 16 mg/L CST for 45 min (Fig. 6B).

### Evaluation of the CST heteroresistance mechanism in ECC

**Fig. 7:**
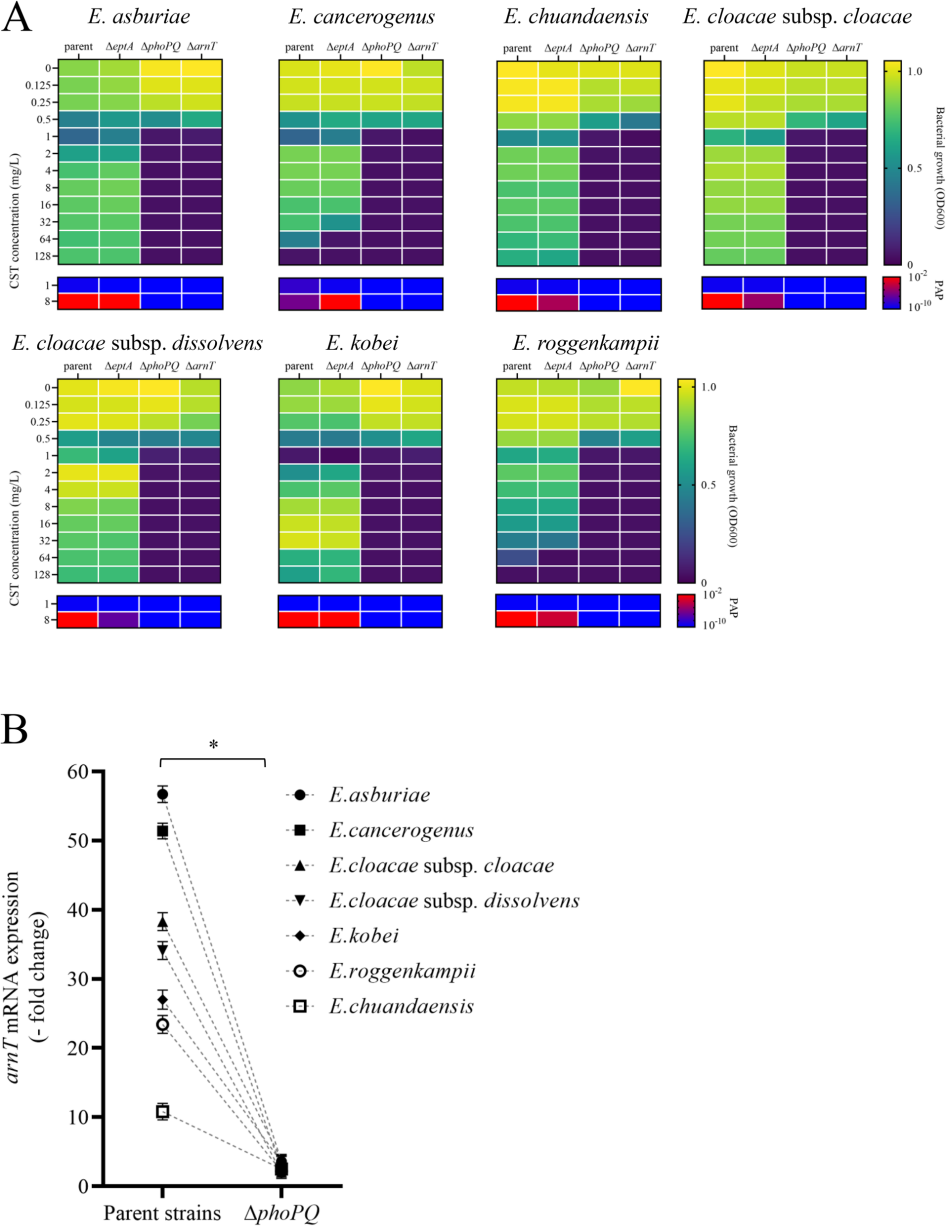
CST-heteroresistant phenotype of *phoPQ-*, *eptA-,* and *arnT-*deleted mutants derived from various species of ECC. Deletion mutants were prepared for the following seven representative species of ECC clinical isolates: SMEnt819 (*E. asburiae* by ANI, *hsp60* cluster I), SMEnt494 (*E. cancerogenus*, *hsp60* cluster I), SMEnt1102 (*E. cloacae* subsp. *cloacae, hsp60* cluster XI), SMEnt835 (*E. cloacae* subsp. *dissolvens*, *hsp60* cluster XII), SMEnt513 (*E. kobei*, *hsp60* cluster II), SMEnt1062 (*E. roggenkampii, hsp60* cluster IV), and SMEnt1003 [*E. chuandaensis* (ANI value 94.1%), *hsp60* cluster IX]. **A** Heat maps representing bacterial growth and occurrence of CST-resistant subpopulations in the presence of CST. Bacterial growth was measured by assessing turbidity (OD_600_) after cultivation for 20 h based on the broth microdilution method in 96-well plates. OD_600_ < 0.1 was defined as no growth. A CST MIC ≥ 4 mg/L was defined as CST resistance. The occurrence frequencies of CST-resistant subpopulations were measured in the presence of 1 or 8 mg/L CST. Color shading was used to visualize the turbidities (OD_600_) and frequencies of CST-resistant subpopulations at each CST concentration. Turbidities (OD_600_) decrease from yellow to purple and occurrence frequencies of CST-resistant subpopulations in PAP decrease from red to blue. **B** Comparison of *arnT* mRNA expression levels in the presence of CST (16 mg/L) between parent strains and their *phoPQ*-deleted mutants. The y axis shows relative mRNA expression levels normalized by those in the absence of CST in each strain (set to 1.0). *p = 0.002

To evaluate the mechanism underlying CST heteroresistance in individual ECC species, we selected one CST-heteroresistant strain from each of the seven species. No mutation involved in CST resistance was found in *phoPQ*, *mgrB*, *pmrAB*, *arnT*, and *eptA* between these CST-heteroresistant and CST-susceptible isolates. We generated *phoPQ-*, *eptA-,* and *arnT*-deleted mutants of the seven isolates. All parent strains and *eptA*-deleted mutants exhibited the typical CST-heteroresistant phenotype in the presence of CST (growth was decreased in the presence of 0.5 or 1 mg/L CST but was observed in the presence of higher CST concentrations) (Fig. 7A). All *phoPQ*- and *arnT*-deleted mutants did not grow in the presence of ≥ 1 mg/L CST. In PAP analysis, the occurrence frequencies of CST-resistant subpopulations were high (> 1 × 10^–3^) in parent strains and *eptA*-deleted mutants in the presence of 8 mg/L CST, but were markedly decreased (< 1 × 10^–8^) and below the criteria of heteroresistance (ranged from 1 × 10^–7^ to 1 × 10^–1^) in *phoPQ*- and *arnT*-deleted mutants (Fig. 7A). In the susceptibility test, the phenotype of *phoPQ*- and *arnT*-deleted mutants changed from CST-resistant to CST-susceptible (Additional file 1: Table S5). Treatment with 16 mg/L CST induced *arnT* mRNA expression more than tenfold in the parent strains of all ECC species, whereas *arnT* mRNA induction by CST was abolished in all *phoPQ*-deleted mutants (Fig. 7B).

**Fig. 8:**
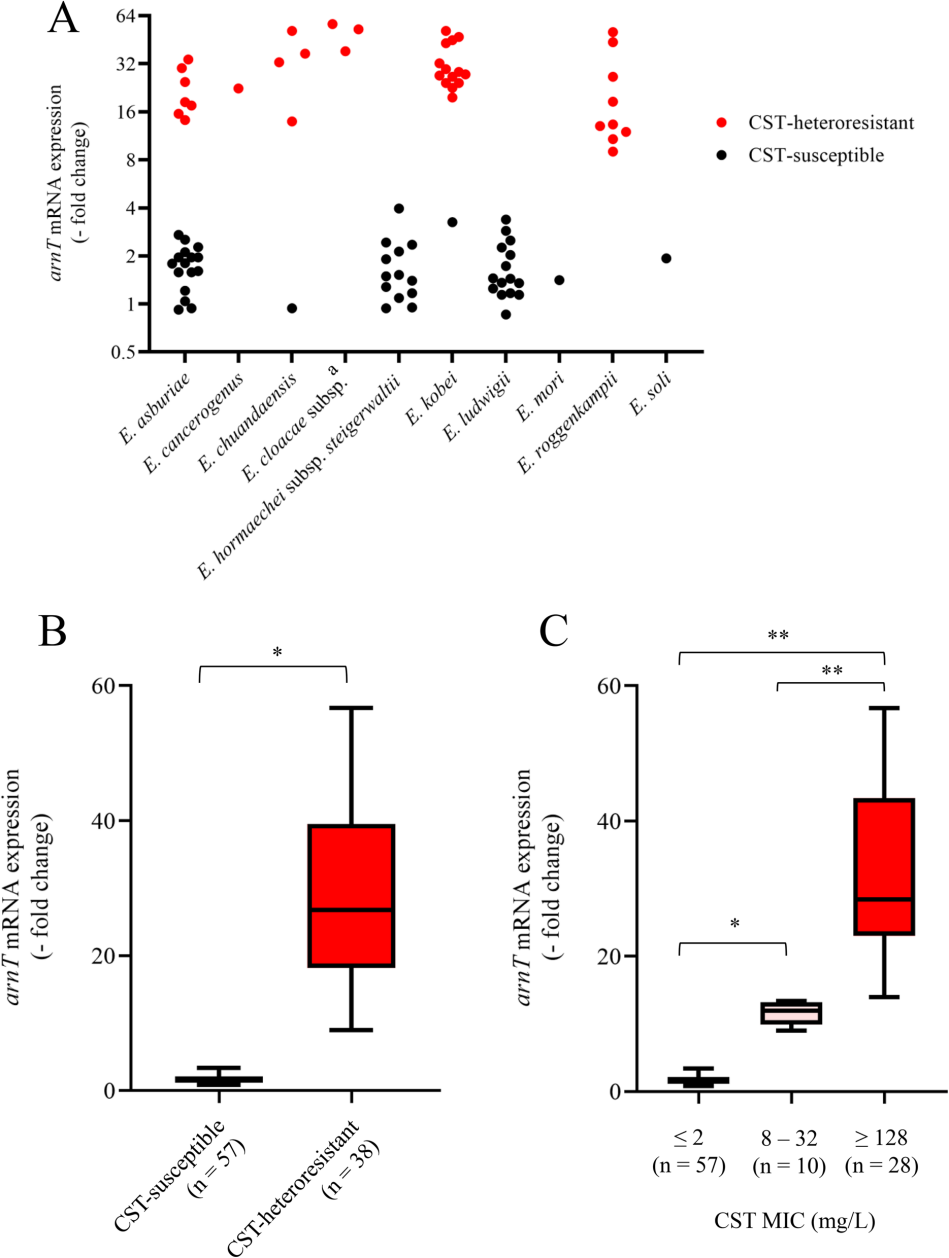
Induction of *arnT* mRNA expression in the presence of CST in ECC. **A** *arnT* mRNA induction by CST in various species found in ECC clinical isolates. The y axis shows relative mRNA expression levels following culture in the presence of 16 mg/L CST for 120 min. The values were normalized by those in the absence of CST in each strain (set to 1.0). CST-heteroresistant and -susceptible isolates are indicated by red and black circles, respectively. Isolates of *E. hormaechei* subsp. *hoffmannii*, *E. hormaechei* subsp. *oharae,* and *E. hormaechei* subsp. *xiangfangensis*, which lack the *arn* operon, were not examined. ^a^, *E. cloacae* subsp. *cloacae* (n = 1) and *E. cloacae* subsp. *dissolvens* (n = 2). **B** Comparison of *arnT* mRNA induction by CST between CST-susceptible and -heteroresistant ECC clinical isolates. The y axis shows relative mRNA expression levels in ECC clinical isolates cultured in the presence of 16 mg/L CST for 120 min. Number in parentheses indicates the number of ECC isolates. The values were normalized by those in the absence of CST in each strain (set to 1.0). *p < 0.001. **C** *arnT* mRNA induction by CST in ECC clinical isolates with low, moderate, and high CST MICs. The y axis shows relative mRNA expression levels in the presence of 16 mg/L CST normalized by those in the absence of CST in each strain (set to 1.0). Number in parentheses indicates the number of ECC isolates. *p < 0.05 and **p < 0.001

In the presence of 16 mg/L CST, *arnT* mRNA expression levels were increased more than eightfold (9.1–56.7-fold) in all CST-heteroresistant isolates, but less than fourfold in CST-susceptible isolates (Fig. 8A, B). The *arnT* mRNA expression levels were positively related with CST MICs (Fig. 8C).

## Discussion

In the present study, 27.5% of ECC clinical isolates obtained from a university hospital in Japan was CST resistant showing heteroresistant phenotype. In the previous studies, the prevalence of CST resistance in ECC clinical isolates was 23.9% in Korea (2012–2014) [11], 11.3–13.3% in UK and Ireland (2017) [10], and 34.5% in China (2020) [34]. These observations indicate that CST-resistant ECC is present in clinical specimens of various origins. Although no multidrug-resistant ECC was detected in our study, the CST resistance in ECC should be an international concern because multidrug-resistant ECC has emerged [9]. However, CST-heteroresistance phenotype has not been adequately examined for the CST-resistant isolates in previous studies [9–11, 16, 17, 34].

The CST-heteroresistant isolates were assigned to certain species and lineages similar to a previous French report [6]. However, no CST-heteroresistant isolates were found in clusters V and VIII in the present study, whereas 100% and 79% of the clusters were CST-heteroresistant in the report, respectively [6] suggesting different CST-heteroresistant lineages have been spread in other counties.

We found several *mcr-9*/*10*-positive isolates in various lineages regardless of CST susceptibility. Acquisition of the plasmid-mediated CST resistance genes *mcr-9* and *mcr-10* contributed little to CST heteroresistance in ECC because CST MICs and occurrence frequencies of CST-resistant subpopulations did not obviously differ between *mcr-9*/*10*-positive and -negative isolates. This observation strongly indicates that CST heteroresistance of ECC was mainly mediated by intrinsic genes, namely, CST-inducible *arnT*, not by exogenous genes.

The STs of CST-heteroresistant isolates were diverse, but *E. asburiae* ST1939 (*hsp60* cluster I), *E. kobei* ST125 (*hsp60* cluster II), and *E. roggenkampii* ST997 (*hsp60* cluster IV) accounted for more than 40% of each species. *E. kobei* ST125 and *E. roggenkampii* ST997 have been clinically isolated in many countries [35–38]. These findings suggest that certain CST-heteroresistant ECC lineages have spread worldwide.

We evaluated the skip-well phenomenon with the broth microdilution method, which is expected to be an easy and useful approach to screen heteroresistant isolates [11]. However, only 26.3% of CST-heteroresistant isolates exhibited the skip-well phenomenon. The isolates with a higher occurrence frequency of CST-resistant subpopulations (from 1 × 10^–4^ to 1 × 10^–2^) by PAP did not display the skip-well phenomenon because this frequency exceeded the inoculating cell number used in the susceptibility test of 5 × 10^5^ CFU/mL (5 × 10^4^ CFU/100 µL in each well of a 96-well plate). Thus, monitoring the skip-well phenomenon was an unreliable method to detect CST heteroresistance in ECC clinical isolates. PAP is a gold standard for detecting heteroresistance [19], but is laborious. We found CST-heteroresistant isolates grew less or not at all at a certain CST concentration (0.5 or 1 mg/L) and grew proficiently at higher CST concentrations. Thus, measuring bacterial growth by assessing turbidity (OD_600_) in the presence of various CST concentrations should be convenient method for screening CST-heteroresistant isolates in routine clinical laboratory. It did not require any additional experiments other than those performed to determine CST MICs by the broth microdilution method (Additional file 1: Table S2).

Why do CST-resistant subpopulations appear? The present study demonstrated that the PhoPQ-*arnT* pathway was involved in CST heteroresistance in certain ECC species, *E. asburiae*, *E. cancerogenus*, *E. chuandaensis*, *E. cloacae* (subsp. *cloacae* and subsp. *dissolvens*), *E. kobei*, and *E. roggenkampii*. Contribution of this pathway has been shown in a reference strain *E. cloacae* subsp. *cloacae* ATCC 13047 [16]. In addition, we revealed that this pathway is activated by CST, and the resulting *arnT* induction occurred in the presence of CST at a concentration around the MIC. Thus, CST-heteroresistant phenomenon, namely decreased bacterial growth in the presence of 0.5 or 1 mg/L CST, was attributed to inadequate (or unresponsive) *arnT* induction at lower CST concentrations (< 2 mg/L), while induced ArnT overcame the bactericidal activity of CST at higher CST concentrations (≥ 2 mg/L). These findings suggest that the appearance of CST-resistant subpopulations in a strain displaying CST heteroresistance reflects the ability of *arnT* induction by CST.

## Conclusion

This study demonstrates that CST-heteroresistant ECC is present in clinical specimens from various infection sites. Inducible CST heteroresistance is mediated via the PhoPQ-*arnT* pathway in CST-resistant isolates of certain ECC species, such as *E. roggenkampii*, *E. kobei*, *E. chuandaensis*, *E. cloacae*, and a part of *E. asburiae*. On the other hand, no CST-resistant isolates were found in *E. hormaechei* and *E. ludwigii*. Therefore, attention is required to be paid to CST heteroresistance at all infection sites of these ECC species, which have a common intrinsic gene background, such as species, *hsp60* clustering, MLST, and/or core genomic features. Exogenous *mcr* genes, such as *mcr-9* and *mcr-10*, found in ECC clinical isolates [34, 39, 40] contribute less to CST resistance.

## Supplementary Information

**Additional file 1: Table S1.** Sequences of primers used in this study. **Table S2.** Characteristics of ECC and *K. aerogenes* clinical isolates used in this study. **Table S3.** Characteristics of CST-heteroresisatnt ECC clinical isolates. **Table S4.** CST MICs of none-*mcr*, *mcr-9*-positive, and *mcr-10*-positive ECC isolates. **Table S5.** Comparison of CST susceptibility of *phoPQ*-, *eptA*-, and *arnT*-deleted mutants of different ECC species.

**Additional file 2: Fig. S1. **Contribution of *mcr-9* and *mcr-10* to CST heteroresistance of ECC clinical isolates. **A** Comparison of the occurrence frequencies of CST-resistant subpopulations among none-*mcr*, *mcr-9*-positive, and *mcr-10*-positive ECC clinical isolates. There was no significant difference among the groups. **B**–**G** Comparison of the occurrence frequencies of CST-resistant subpopulations between *mcr-9*-positive and -negative **B**–**D** and between *mcr-10*-positive and -negative **E**–**G** isolates. These isolates were selected from the same node of the core genome phylogeny. PAP was performed with the following CST concentrations: 0.125 (**B** and **E**), 1 (**C** and **F**), and 8 (**D** and **G**) mg/L. There were no significant differences among the groups. ^a^, *E. hormaechei* subsp. *xiangfangensis*.

## Data Availability

Nucleotide sequence data reported are available in the DDBJ/EMBL/GenBank databases under accession numbers from DRX398430 to DRX398524.

## Abbreviations

ANI: Average nucleotide identity
CLSI: Clinical and Laboratory Standards Institute
CST: Colistin
ECC: *Enterobacter cloacae* complex
*hsp60*: Heat shock protein 60 gene
L-Ara4N: 4-Amino-4-deoxy-l-arabinose
MALDI-TOF MS: Matrix-assisted laser desorption/ionization time-of-flight mass spectrometry
MHII agar: Mueller–Hinton II agar
MHII broth: Mueller–Hinton II broth
MIC: Minimum inhibitory concentration
MLST: Multilocus sequence typing
PAP: Population analysis profiling
pEtN: Phosphoethanolamine
RT-qPCR: Reverse transcription quantitative PCR
SNPs: Single nucleotide polymorphisms
ST: Sequence type
TSB: Trypticase soy broth
WGS: Whole-genome sequencing
