# Supplementary figures and images for: High prevalence of colistin heteroresistance in specific species and lineages of Enterobacter cloacae complex derived from human clinical specimens
Source: Ann Clin Microbiol Antimicrob. 2023 Jul 15;22:60. doi: 10.1186/s12941-023-00610-1 (PMC10350281; doi:10.1186/s12941-023-00610-1)

## Slide 1
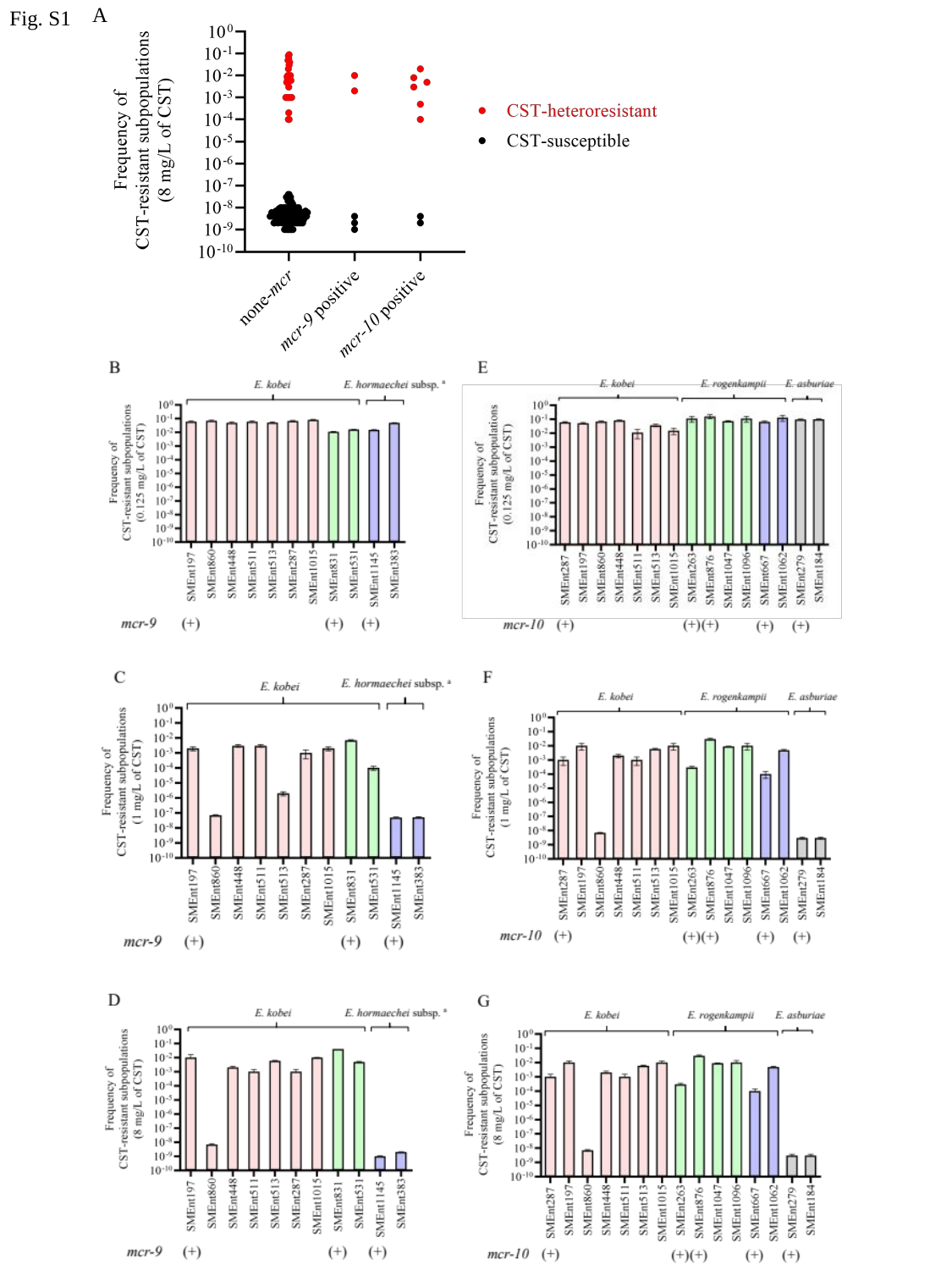

A
Fig. S1

Supplement: Supplementary file 2 — Additional file 2: Fig. S1. Contribution of mcr-9 and mcr-10 to CST heteroresistance of ECC clinical isolates. A Comparison of the occurrence frequencies of CST-resistant subpopulations among none-mcr, mcr-9-positive, and mcr-10-positive ECC clinical isolates. There was no significant difference among the groups. B–G Comparison of the occurrence frequencies of CST-resistant subpopulations between mcr-9-positive and -negative B–D and between mcr-10-positive and -negative E–G isolates. These isolates were selected from the same node of the core genome phylogeny. PAP was performed with the following CST concentrations: 0.125 (B and E), 1 (C and F), and 8 (D and G) mg/L. There were no significant differences among the groups. a, E. hormaechei subsp. xiangfangensis. [file 12941_2023_610_MOESM2_ESM.pptx]
